# The characteristics and efficacy of catheter ablation of focal atrial tachycardia arising from an epicardial site

**DOI:** 10.1002/clc.23577

**Published:** 2021-02-18

**Authors:** Teppei Yamamoto, Yu‐ki Iwasaki, Yuhi Fujimoto, Eiichiro Oka, Hiroshi Hayashi, Hiroshige Murata, Kenji Yodogawa, Meiso Hayashi, Osamu Igawa, Wataru Shimizu

**Affiliations:** ^1^ Department of Cardiovascular Medicine Nippon Medical School Tokyo Japan

**Keywords:** ablation, atrial tachycardia, autonomic nerve activity, epicardial adipose tissue, respiratory, swallowing

## Abstract

**Background:**

Although epicardial structures around the atrium such as adipose tissue possess arrhythmogenicity, little is known about atrial tachycardias (ATs) originating from epicardial sites (Epi‐ATs). This study aimed to elucidate the prevalence, characteristics, and outcome after radiofrequency catheter ablation (RFCA) of Epi‐ATs and to reveal the association between Epi‐ATs and the epicardial structures.

**Methods:**

The electrocardiographic, electrophysiologic, and anatomical properties and results of RFCA were analyzed in 42 patients with a total of 49 ectopic ATs.

**Results:**

Six Epi‐ATs (12%) were observed in six patients (14%). Four of six were respiratory cycle‐dependent ATs and one was a swallowing‐induced AT. The Epi‐AT origins were adjacent to a pulmonary vein (five cases) and vein of Marshall (one case). A Valsalva maneuver or atropine infusion to define the arrhythmia mechanism affected the appearance of the Epi‐ATs. The congruity rate between epicardial adipose tissue and the AT origin was significantly higher (100% vs. 44%, *p* = .045), and the epicardial adipose tissue volume of the atrium was significantly larger (104.1 vs. 64.6 ml, *p* = .04) in the Epi‐AT group. Endocardial RFCA targeting the AT foci resulted in acute success in five of five cases. However, electrical isolation including of the AT foci resulted in acute failures (two of three cases) or a recurrence (one of one case).

**Conclusions:**

Six Epi‐ATs were associated with thoracic veins and epicardial arrhythmogenic structures. The main cause provoking the Epi‐ATs was associated with autonomic nerve activity.

## 1 INTRODUCTION

The epicardial structures in the atrium such as the coronary sinus (CS), ligament of Marshall (LOM), and epicardial adipose tissue are associated with atrial arrhythmogenicity.^1^, ^2^, ^3^, ^4^, ^5^ The mechanism has not been fully clarified and is considered multifactorial. One factor is that these are autonomic nerve rich structures and include ganglionated plexi (GPs). Post ganglionic efferent fibers innervate the atrial myocardium and can provoke supraventricular arrhythmias especially atrial fibrillation.^1^ Other earlier studies^2^, ^3^, ^4^, ^5^ have reported the participation of chronic low‐grade inflammation, oxidative stress, and the formation of reentrant circuits from intramyocardial fat and via the muscular bundles of the LOM and/or CS. On the other hand, focal atrial tachycardias (ATs) arising from the epicardial side (Epi‐AT) are rare arrhythmias or may be underestimated or unrecognized during catheter ablation (CA), because Epi‐ATs can often be successfully treated with a similar procedure as that used in ablation of ordinary ectopic ATs, and because such ATs may be recognized as unmappable ATs during endocardial CA. Only a few Epi‐AT cases using the percutaneous epicardial approach or high density contact mapping approach have been demonstrated and successfully ablated.^6^, ^7^, ^8^ At present, little is known about the clinical and electrophysiologic characteristics of Epi‐ATs. Therefore, we investigated the incidence and the electrophysiologic properties of Epi‐ATs, their origins, effects of drugs and the Valsalva maneuver, and the efficacy of CA, along with the clinical background in patients with Epi‐ATs. The clinical course of Epi‐ATs and their outcomes after CA were also compared with other focal non‐Epi‐ATs.

## 2 METHODS

### 2.1 Study population and definition of Epi‐AT and other terms

From April 2016 to April 2018, 42 consecutive patients with a total of 49 focal ATs were referred to the Nippon Medical School Main Hospital and underwent a radiofrequency (RF) CA. Written informed consent was obtained, and this study was approved by the ethics committee of Nippon Medical School. We analyzed the clinical characteristics of the patients, electrocardiographic and electrophysiology properties of the ATs, and results of the CA. For each patient, the data were obtained from the patient chart, body surface electrocardiograms before, during and after the electrophysiology study, 24‐hour Holter monitoring, echocardiography, and intracardiac electrograms during the electrophysiology study. Electroanatomical mapping data were also analyzed. We defined an Epi‐AT as an AT which had multiple earliest activation sites (EASs) on the endocardial activation map or which had a centrifugal activation pattern and could be eliminated by RF applications at remote epicardial regions from the EASs. The mechanism of respiratory cycle‐dependent AT (RCAT) and swallowing‐induced AT (SIAT) has been considered to be related to GPs including epicardial adipose tissue.^9^, ^10^ An RCAT was defined as an AT that occurred after initiating inspiration and ceased during the following expiration during a minimum of five consecutive respiration cycles.^9^ SIAT was defined as an AT that occurred only after swallowing.

### 2.2 Pharmacological properties

Before or during the electrophysiology study, some drugs (isoproterenol, adenosine‐triphosphate, and atropine) were administered to determine the mechanism of the Epi‐ATs. Isoproterenol (0.005–0.01 mg/kg^−1^/h^−1^) was administered during and after the EP study to observe the appearance, inducibility, or persistence of the Epi‐ATs. An adenosine‐triphosphate bolus infusion was performed for observing whether the Epi‐ATs transiently terminated after the infusion. In the case that an RCAT or SIAT was detected before the EP study, an atropine (0.5 mg) bolus infusion was performed to observe the appearance or persistence of these ATs.

### 2.3 Electrophysiologic study, mapping of tachycardias, and radiofrequency ablation

The study was performed in the fasting state, with unconscious sedation using dexmedetomidine. All antiarrhythmic drugs were discontinued for a minimum of five half‐lives before the procedure. An electroanatomical mapping system was used in all patients: a CARTO system (Biosense Webster Inc., Diamond Bar, CA) or EnSite Velocity system (Velocity, Abbott, Abbot Park, IL). A 20‐polar catheter with 2–2–2 mm interelectrode spacing (BeeAT, Japan Lifeline Co., Ltd, Tokyo, Japan) was introduced from the right internal jugular vein and advanced into the CS. An irrigated RF ablation catheter (SmartTouch, Biosense Webster Inc. or FlexAbility, Abbott), which was also used as a mapping catheter with the electroanatomical mapping system, was introduced into the atrium. In patients who required isolation of the pulmonary veins (PVs), a 20‐pole circumferential catheter (Lasso, Biosense Webster or Optima, Abbott) and/or a 20‐pole mapping catheter (PentaRay, Biosense Webster) was located at the ostium of the target vein and was used to create the electroanatomical map. Body surface ECGs and bipolar endocardial electrograms were monitored continuously and recorded with an EP‐WorkMate (Abbott) recording system at a filter setting of 30–500 Hz. Bipolar pacing was performed using an EP MedSystems programmable stimulator. In patients in whom spontaneous AT did not emerge, programmed atrial stimulation was delivered using burst pacing or an eight‐stimulus drive train followed by single or double extrastimuli from the CS with and without an isoproterenol infusion. The anatomical localization of the atrial focus was accomplished during the tachycardia by the analysis of the high density atrial activation using a 20‐pole circumferential and/or PentaRay catheter with electroanatomical mapping system.^11^, ^12^ When the tachycardia was considered to have a left‐sided origin, a trans‐septal puncture using conventional techniques with the use of a long vascular sheath was performed. When the tachycardia was considered to be related to the Marshall bundle, we cannulated the vein of Marshall (VOM) with a 2 Fr octa‐polar electrode catheter (EP star Fix, Japan Lifeline) through the lumen of a deca‐polar catheter (Response, Abbott). RF energy was delivered between the distal electrode of the ablation catheter and a cutaneous adhesive electrode on the lower trunk using a RF generator (Stockert J70 RF Generator, Stockert GmbH, Freiburg, Germany, or Ampere RF Ablation Generator, Abbott). RF energy was delivered for 20–60 seconds. The temperature control mode was limited to 38°C and a maximum power of 40 W. Acute success of the ablation procedure was defined as the absence of any spontaneous or induced AT by programmed stimulation with and without an isoproterenol infusion (0.005–0.01 mg/kg^−1^/h^−1^) for at least 30 minutes after the ablation.

### 2.4 Measurement and analysis of epicardial adipose tissue

The epicardial adipose tissue volume was calculated from contrast images obtained with a 3D spiral computed tomography (CT) scanner (320‐row detector, dynamic volume CT scanner; Aquilion ONE, Toshiba Medical Systems, Tokyo, Japan) before the radiofrequency catheter ablation (RFCA). The data transferred to the EnSite Verismo segmentation tool (Velocity, Abbott, Abbott Park, IL) was used for the analysis of the epicardial adipose tissue. The CT value threshold was set between −50 and −200 Hounsfield units to detect the epicardial adipose tissue. The volume of the epicardial adipose tissue surrounding each chamber was manually segmented from the total epicardial adipose tissue.^13^ The segmented planes of each chamber were overlayed on the mitral annulus and tricuspid annulus in order to separate them between the atrium and the ventricle and on the connected plane between the anterior antrum of the right PV and ostium of the CS to separate both atria. These CT images were merged or compared with the 3D electroanatomical maps. The CT images were analyzed by 1 electrophysiologist and 1 clinical engineer in a blinded manner.

### 2.5 Statistical analysis

The data were expressed as the mean ± SD for continuous variables and as the frequency (number [%]) for categorical variables. For the continuous variables, the differences between groups were compared using the Mann–Whitney *U*‐test and Student's *t*‐test. Because the results were similar, only the latter are presented. For categorical variables, the differences between groups were compared using a Fisher exact test. The correlation coefficient was determined by linear regression analysis. All tests were two‐sided, and a *p* < .05 was considered significant. All statistical analyses were conducted using EZR software^14^ (Saitama Medical Center, Jichi Medical University, Saitama, Japan), which is a convenient user interface for R (The R foundation for Statistical Computing, Vienna, Austria).

## 3 RESULTS

### 3.1 Clinical characteristics of Epi‐ATs and a comparison of Epi‐ATs and non‐Epi‐ATs

Among the 42 patients with a total of 49 non‐reentrant focal ATs, six distinct Epi‐ATs (12%) were found in six patients (14%) (AT‐1 to −6: four males and two females) with a mean age of 61 ± 7 [55–76] years‐old. The clinical characteristics of the patients with Epi‐ATs and non‐Epi‐ATs are shown in Table 1. The age was significantly younger and prevalence of RCATs significantly higher in the Epi‐AT group, while no other significant differences were confirmed in terms of the clinical characteristics between the patients in the two groups. The successful ablation sites of the six Epi‐ATs were compared with the 43 focal non‐Epi‐ATs. The successful ablation site was at the antrum of the right PV in three Epi‐ATs and three non‐Epi‐ATs (50% vs. 7%, *p* = .02), or at the LA posterior roof close to the left PV in two Epi‐ATs and no non‐Epi‐ATs (33% vs. 0%, *p* = .01). The clinical characteristics of the six Epi‐ATs are presented in Table 2. There was no detectable structural heart disease in any of the Epi‐AT patients. In five patients (AT‐1, −2, −3 ‐5, and −6), the ATs were of an incessant form (4 RCATs and 1 SIAT). The average coupling interval of 100 RCAT and SIAT events (analysis of 20 events in each RCAT and SIAT) was 303 ± 46 ms. All RCATs and 1 SIAT were irregular, and the mean AT cycle length varied among the ATs, ranging from 265 to 358 ms. AT‐4 showed a persistent AT with a regular tachycardia cycle length of 450 ms. In that case, we performed post pacing interval mapping and there were no sites remote from the EAS where the return cycle after entrainment pacing was identical to the tachycardia cycle length because of the exclusion of macro‐reentrant ATs such as a Marshall bundle reentry.^15^ In AT‐1, AT‐2, AT‐5, and AT‐6, 3 RCATs and 1 SIAT, were suppressed during and shortly after (two or three respiratory cycles) a Valsalva maneuver during the end‐inspiratory phase (see the Video S1).

**TABLE 1:** Comparison of the characteristics between the patients with an Epi‐AT and those with a non‐Epi‐AT

	Total (42 patients, 49 ATs)	Epi‐AT (six patients, six ATs)	Non‐Epi‐AT (36 patients, 43 ATs)	*p* value
Age, years	68 ± 11	61 ± 7	69 ± 11	.03
Male gender, *N* (%)	22 (52)	4 (67)	18 (50)	.67
BMI	23 ± 4	25 ± 4	22 ± 3	.11
RCAT	6 (12)	4 (67)	2 (5)	.002
Recurrence of AT, *N* (%)	11 (22)	1 (17)	10 (23)	1.00
P wave duration during AT (ms)	80 ± 20	89 ± 22	78 ± 19	.22
AF, *N* (%)	27 (64)	4 (67)	23 (64)	1.00
Persistent AF, *N* (%)	13 (31)	1 (25)	12 (33)	.60
Recurrence of AF, *N* (%)	4 (10)	0	4 (13)	.56
Structural heart disease, *N* (%)	6 (14)	0	6 (17)	.57
Hypertensive heart disease	2 (5)	0	2 (6)	1.00
TCM	1 (2)	0	1 (3)	1.00
Other cardiomyopathies	3 (7)	0	3 (8)	1.00
Follow‐up period, month	13 ± 7	13 ± 8	13 ± 7	.98
Echocardiographic parameters				
LV ejection fraction, %	66 ± 11	69 ± 4	65 ± 12	.45
LA dimension, mm	38 ± 8	38 ± 7	38 ± 8	.95

Abbreviations: AF, atrial fibrillation; AT, atrial tachycardia; BMI, body mass index; Epi‐AT, epicardial origin atrial tachycardia; RCAT, respiratory cycle‐dependent atrial tachycardia; TCM, tachycardia‐induced cardiomyopathy.

**TABLE 2:** The clinical and electrophysiologic properties of the Epi‐ATs

AT	Age, years	Sex	Earliest activation site (M = multiple sites)	Mean tachycardia cycle length (range), ms	Ablation site or target	Efficacy of electrical isolation of AT	RF power required to eliminate, W	Form of AT	GP reaction during successful RF application	Fractionated potential/abnormal voltage at the successful site
1	58	M	LA roof (M)	275 (262–298)	LA posterior wall isolation	Acute success but recurrence	40	RCAT	SB	+/‐
2	60	F	LA inferior (M), VOM	358 (295–449)	Marshall bundle	NA	35	RCAT	‐	‐/‐
3	55	M	LA posteroinferior (M)	265 (231–315)	AT focus or ARGP	NA	30	RCAT	‐	+/‐
4	76	F	SVC‐RA connection	450 (NA)	AT focus or ARGP	Acute unsuccess	35	Regular AT	SB	+/+
5	58	M	LA posteroinferior	308 (268–340)	AT focus or ARGP	NA	30	RCAT	SB	‐/‐
6	56	M	LSPV (M)	309 (278–340)	AT focus or SLGP	Acute unsuccess	40	SIAT	SB	+/‐

AT	Preceded time from the P wave at the success site, ms	Time between the EAS to success site, ms	Pre‐applied drugs and effects to the Epi‐AT	
1	NA (electrical isolation)	NA (electrical isolation)	Bisoprolol 2.5 mg, Flecainide 200 mg	NA, NA
2	5	5	Bisoprolol 2.5 mg	No
3	40	30	NA	NA
4	NA (electrically isolated)	NA (electrically isolated)	Pilsicainide 150 mg, Sotalol 120 mg	NA, NA
5	−10	70	Pilsicainide 150 mg, Atropine 0.5 mg	no transiently suppressed
6	50	5	Flecainide 150 mg, Atropine 0.5 mg	no, transiently suppressed

Abbreviations: ARGP, anterior right ganglionated plexi; AT, atrial tachycardia; EAS, earliest activation site; Epi‐AT, epicardial origin atrial tachycardia; GP, ganglionated plexi; RCAT, respiratory cycle‐dependent atrial tachycardia; SB, sinus bradycardia; SIAT swallowing induced atrial tachycardia; SLGP superior left ganglionated plexi; SVC superior vena cava.

### 3.2 Pharmacological properties

The Epi‐ATs were refractory to 1.2 ± 0.4 drugs including class I and class III antiarrhythmic agents and beta blockers. Isoproterenol and 10 mg of an adenosine‐triphosphate infusion during the electrophysiologic study did not affect the inducibility or persistence of the Epi‐ATs in all patients. A 0.5 mg atropine infusion transiently suppressed the Epi‐ATs in AT‐5 and AT‐6.

### 3.3 Mapping and outcomes of catheter ablation in patients with Epi‐ATs

**FIGURE 1:**
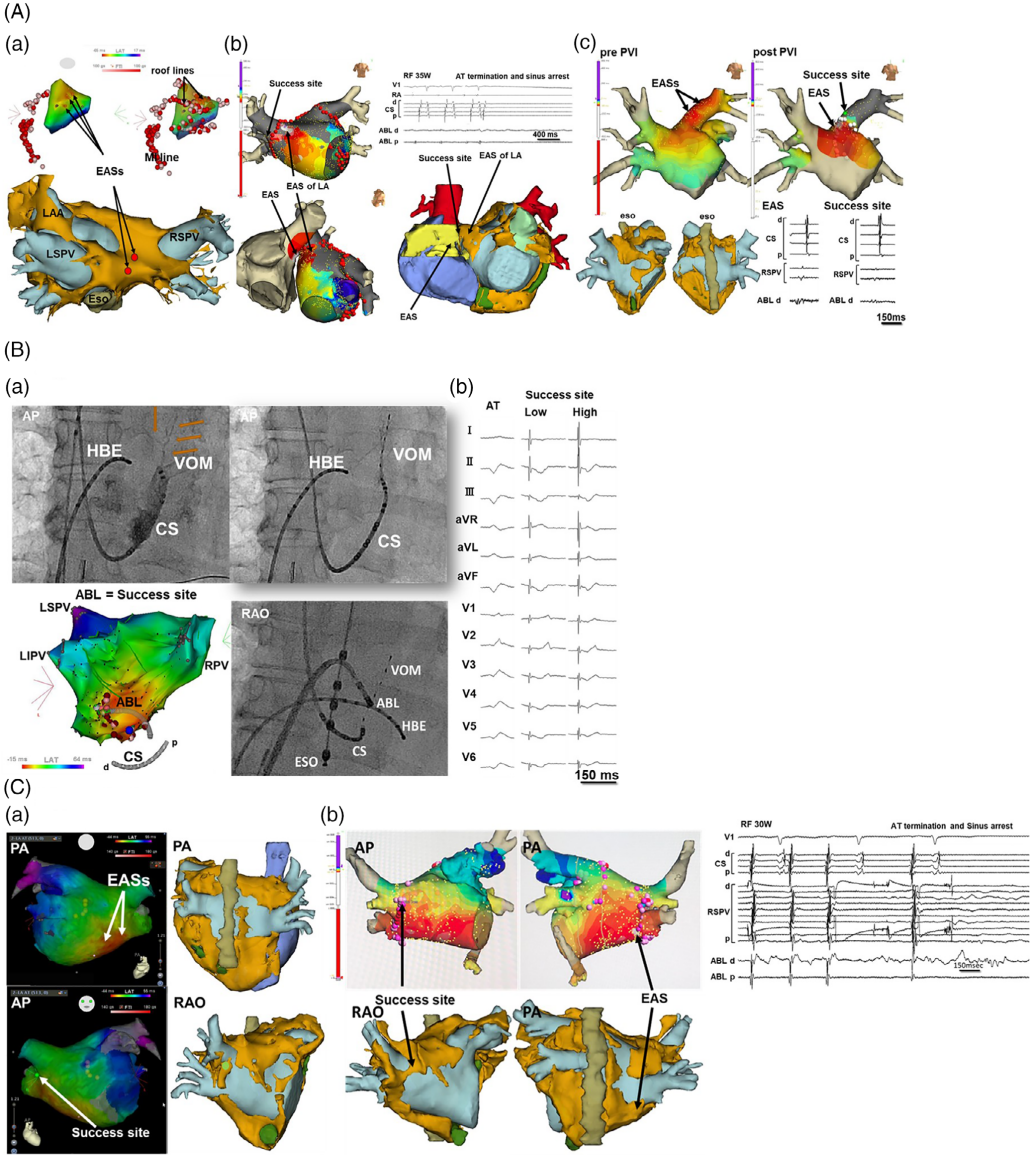
(A) The electroanatomical maps and CT images of the atrium during AT‐1 (a), AT‐4 (b), and AT‐6 (c), respectively. The light brown structure in the CT images was epicardial adipose tissue. These three atrial tachycardias were eliminated by the electrical isolation or radio frequency (RF) applications in the electrically isolated region. (a) Although the radio frequency applications at the earliest activation site (EASs) were not effective, electrical isolation of the LA posterior wall eliminated AT‐1. (b) The activation map in the LA (b‐upper left) showed that the EAS in the LA was adjacent to the right superior PV (RSPV) isolation line. The actual EAS was at the junction between the superior vena cava and right atrium (b‐lower left). The successful application was obtained at a site with no recordable potentials (b‐upper right). (c) The activation map revealed multiple EASs at the left superior PV (LSPV, c‐upper right). A left PV isolation resulted in the movement of the EAS 1 cm away from the isolation line. Only small far field potentials at the successful site were recorded (c‐lower right). CS: coronary sinus, Eso: esophagus, LAA: left atrial appendage, MI: mitral isthmus. (B) The electroanatomical map and coronary sinus (CS) angiogram of AT‐2 showing that the vein of Marshall (VOM, arrows) was detected at a site opposite the earliest site (a‐left). We cannulated the VOM with a 2 Fr octapolar catheter (a‐upper left) and ablated that site (a‐lower left). We also performed pace‐mapping, which showed that the P‐wave morphology during high output pacing at the endocardial success site was only identical to that of AT‐2 (b). ABL: ablation catheter, AT: atrial tachycardia, Eso: esophageal catheter, HBE = His bundle electrode, LIPV: left inferior pulmonary vein, LSPV: left superior pulmonary vein, RPV: right pulmonary vein. (C) The activation maps and CT images of AT‐3 (a) and AT‐5 (b) in the LA, respectively. The elimination pattern of the tachycardias showed that the successful sites were separate from the earliest activation sites (EAS). The successful sites of both tachycardias were at the antrum of the anterior right superior pulmonary vein, which was a ganglionated plexus rich region. Indeed, epicardial adipose tissues existed at the successful sites. RF: radio frequency

**FIGURE 2:**
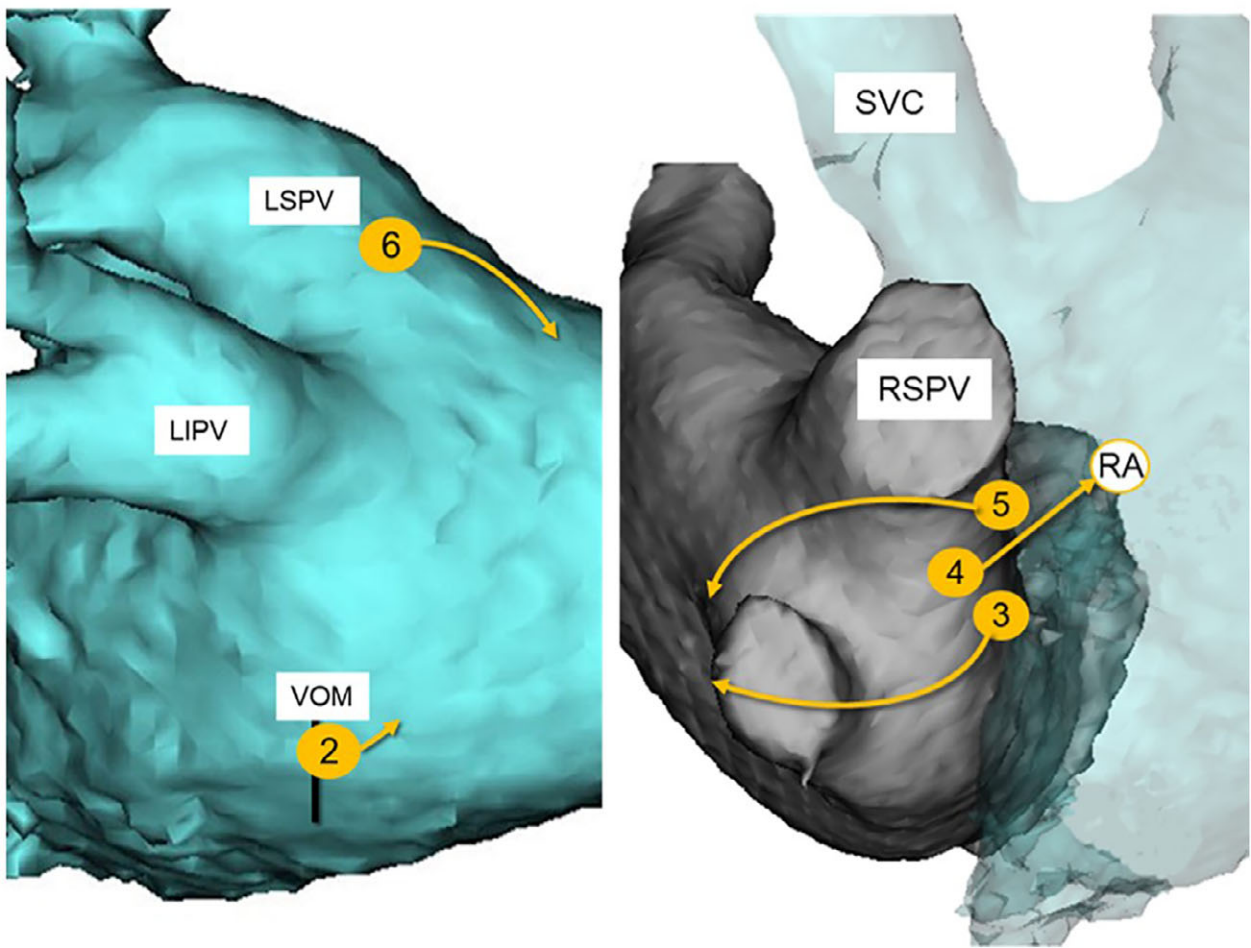
The origins of each Epi‐AT were marked on CT images (number in circle). Arrow heads indicated each earliest activation sites in endocardial activation maps. LIPV: left inferior pulmonary vein, LSPV: left superior pulmonary vein, RSPV: right superior pulmonary vein, SVC: superior vena cava, VOM: the vein of Marshall

The bipolar electrogram at the EASs preceded the onset of the P wave by 39 ± 18 ms (10–70). The electroanatomical maps and CT images of each Epi‐AT case are presented in Figures 1 and 2. In AT‐4, the electroanatomical map (Figure 1(A)‐(b)) from the endocardium exhibited a centrifugal activation pattern and the EAS was located at the connection between the RA and SVC. The EAS and successful site were anatomically separate from each other, and the successful site was electrically isolated the anterior right PV antrum. In AT‐1, AT‐2, AT‐3, and AT‐6, there were multiple EASs on the endocardial activation maps at the LA posterior roof close to the left PV, LA inferior, LA posteroinferior adjacent to the right inferior PV, LA posteroinferior adjacent to the right inferior PV, and the left superior PV, respectively. In AT‐2, the actual EAS was located in the VOM opposite the EAS on the endocardial map (Figure 1(B)‐(a)). A pacemap was performed because the EAS was detected at an epicardial site during the AT. The P wave morphology was only identical at the successful site with high amplitude pacing (Figure 1(B)‐(b)). A focal RF delivery on the endocardial side of the LA that was opposite the actual EAS in the VOM successfully eliminated AT‐2. Electrical isolation of the left posterior wall including the EASs was performed in AT‐1, which resulted in the complete suppression of the Epi‐AT with no ectopic beats inside the posterior wall (Figure 1(A)‐(a)). On the other hand, an extensive left PV isolation including of the EASs was not effective in AT‐6 (Figure 1(A)‐(c)). After the left PV isolation with complete exit block, the EAS was displaced to outside the isolation line. Although an RF application at the displaced EAS could not eliminate AT‐6, AT‐6 was successfully ablated at the site of the isolated left superior PV antrum, which was located 1 cm from the isolation line. In AT‐3 and AT‐5, a successful ablation was achieved during a right PV isolation of AF (still not isolated) at the anterior aspect of the right superior PV antrum, which was at least 2 cm from each EAS (Figure 1(C)). In AT‐4 and AT‐5 the ablation sites after unsuccessful applications at the EASs and/or electrical isolation were determined due to the experience in AT‐3. In AT‐3, after an unsuccessful RF application at the EAS, we performed a right PV isolation of the AF and could unexpectedly eliminate AT‐3 at the anterior right superior PV antrum, in which the anterior right GPs were often located. Parasympathetic nerve responses during and shortly after the successful RF application such as a sinus pause or atrioventricular block were observed in AT‐1, AT‐4, AT‐5, and AT‐6. Fractionated potentials and an abnormal voltage during the AT or sinus rhythm at the success sites were recorded in four of six and one of six Epi‐AT cases, respectively. Acute success during the first ablation procedure was achieved in all patients. There were no major complications during or after the ablation procedures in all cases. During the follow‐up period of 13 ± 8 months (range 4 to 29 months), no ATs were observed in any of the patients without any antiarrhythmic drug use except for AT‐1 in which the Epi‐AT recurred 1 day after the procedure.

### 3.4 Relationship between the special distribution of epicardial adipose tissue and Epi‐ATs

Cardiac CT images were obtained in five of the six Epi‐AT patients (Figure 1) and 16 of 36 non‐Epi‐AT patients: four of four AF cases and one SIAT case in the Epi‐AT group and 16 of 23 AF cases in the non‐Epi‐AT group. The clinical characteristics and analysis data of epicardial adipose tissue between the Epi‐AT and non‐Epi‐AT groups are shown in Table 3. In all Epi‐AT cases, there was epicardial adipose tissue at the Epi‐AT origin and EASs, and in contrast, in only seven of 16 cases in the non‐Epi‐AT group (100% vs. 44%, *p* = .045). Cardiac CT also showed that the epicardial adipose tissue volume of the total atrium was significantly larger in the Epi‐AT group (104.1 vs. 64.6 ml, *p* = .04), while the LA and total atrium volumes did not significantly differ. On the other hand, the total adipose tissue volume around the atrium was significantly correlated with the body weight (*p* = .04, *r* = 0.54) as in a previous report.^13^ No other clinical characteristics were correlated or had any statistical differences with the presence of adipose tissue in this study.

**TABLE 3:** The analysis of the relationship between AT and epicardial adipose tissue

	Total (21 patients, 21 ATs)	Epi‐AT (five patients, five ATs)	Non‐Epi‐AT (16 patients, 16 ATs)	*p* value
Age, years	69 ± 8	61 ± 8	71 ± 6	.005
Male gender, *N* (%)	11 (52)	4 (80)	7 (44)	.17
BMI	24 ± 4	26 ± 3	23 ± 4	.07
RCAT, *N* (%)	4 (19)	3 (60)	1 (6)	.01
AF, *N* (%)	20 (95)	4 (80)	16 (100)	.24
Persistent AF, *N* (%)	8 (38)	1 (20)	7 (44)	.62
Recurrence of AF, *N* (%)	3 (14)	0 (0)	3 (19)	1.00
Follow‐up period, month	14 ± 7	13 ± 8	15 ± 7	.76
Computed tomography image analysis				
Congruity to AT focus, *N* (%)	12 (57)	5 (100)	7 (44)	.045
LA volume, ml	107.8 ± 34.9	98.1 ± 30.8	110.9 ± 35.5	.5
RA volume, ml	126.6 ± 58.5	130.2 ± 27.9	125. 5 ± 65.2	.88
EAT volume, ml				
Total atrium	74.0 ± 36.6	104.1 ± 30.4	64.6 ± 33.1	.04
LA	42.4 ± 18.9	63.1 ± 13.1	35.9 ± 15.5	.003
RA	31.6 ± 22.1	41.0 ± 25.8	28.7 ± 20.0	.3

Abbreviations: AF, atrial fibrillation; AT, atrial tachycardia; BMI, body mass index; EAT, epicardial adipose tissue; Epi‐AT, epicardial origin atrial tachycardia; RCAT, respiratory cycle‐dependent atrial tachycardia.

## 4 DISCUSSION

The present study reported that, among 49 non‐reentrant focal ATs in 42 patients, six Epi‐ATs (12%) were observed in six patients (14%). These arrhythmias were refractory to antiarrhythmic drugs and beta‐blockers. Five of six arrhythmias developed with an incessant form and the tachycardia cycle length was irregular and varied among the Epi‐ATs.

To date, only three reports of four Epi‐AT cases have been published. Phillips et al.^6^ and Yamada et al.^8^ demonstrated three juvenile cases with Epi‐ATs that were successfully treated by percutaneous pericardial CA. Another report,^7^ exhibiting a juvenile incessant form of an Epi‐AT showed that the tachycardia was diagnosed by endocardial high density mapping and epicardial noninvasive electrocardiographic imaging and that the Epi‐AT was successfully treated at the endocardial breakout sites. These reported Epi‐ATs, all of which were located around the LA appendage, were considered to be related to the Marshall bundle.^7^ However, these reports did not reveal the etiology, characteristics, and mechanisms of the ATs. To the best of our knowledge, the present study is the first report the demonstration of the characteristics and etiology of Epi‐ATs, especially in relation to the autonomic nervous system and epicardial structures.

There were three elimination patterns of the Epi‐ATs. The first was an extensive electrical isolation including of the EASs (Figure 1(A)). The second was that RF energy from the endocardium directly affected the AT origin, and the EAS was determined to be located on the epicardial side (Figure 1(B)). The third was that the RF energy also directly affected the AT origin but the endocardial EAS was not observed at the successful site (Figure 1(C)). These patterns lead to the hypothetical mechanism of the Epi‐ATs: the origin of the Epi‐AT was an epicardial muscle bundle and there were epicardial pathways bridging from the origin to the endocardial breakthrough sites such as the preferential pathway in patients with premature ventricular beats. Previous reports showed that the Marshall bundle had multiple connections with the LA myocardium and formed an arrhythmogenic substrate.^3^, ^15^, ^16^ Barrio‐Lopez et al.^16^ revealed the existence of epicardial electrical connections between the PVs and other structures and that the existence of these veno‐atrial epicardial connections made the PV isolation more difficult and worsened the isolation durability. This existence of epicardial connections was also reported to be a higher risk of atrial tachyarrhythmia recurrence after a PV circumferential isolation.^16^ Other reports^17^, ^18^ showed that there were interatrial epicardial connections between the right‐sided PV antrum and RA. Miyazaki et al.^18^ reported that a focal AT arising from the right superior PV antrum broke though the RA. In that case, the pseudo EAS in the RA was anatomically separate from the actual origin as well as in our cases. In AT‐4 and AT‐6, the difference from that case was that the RF applications at the earliest activation site in the RA and/or LA were not effective, but the successful site was localized inside the electrically isolated PV antrum with exit block (Figure 1(C)). These facts certified that there are epicardial preferential pathways and the ATs originated from the epicardial foci.

The other possibility of the Epi‐AT mechanism was that GP activity directly provoked the Epi‐ATs via their axons, which are distributed in the myocardium at the EASs. Previous canine experimental models^19^ showed that focal AT was induced by GP stimulation. Another study^1^ in silico for simulating the human heart demonstrated that GP stimulation, both sympathetic and parasympathetic, induced a spontaneous phase 3 early after‐depolarization like the action potentials and PV tachycardias adjacent to the stimulated GPs. Several observations in the present study suggested that the autonomic nervous system network was considered to play a major role in the provoking mechanism of Epi‐ATs. First, in five of six patients, their Epi‐ATs were RCATs or SIATs of which the main part of the mechanism was considered to be autonomic nervous irritation.^9^, ^10^ Second, in four of four patients, the Epi‐ATs were transiently suppressed by a Valsalva maneuver with a deep inspiration position. Suppressing arrhythmias with deep inspiration also denies the mechanical stretch theory^9^, ^20^ in RCAT patients. Third, a bolus infusion of atropine transiently terminated two of two ATs. Fourth, in all Epi‐AT patients, there were GP rich epicardial structures, which included epicardial adipose tissue or the LOM at the EASs and successful sites, and in four of six patients, successful RF applications provoked a GP reaction. Thus, autonomic nervous activity is considered to be the main part of the provocation etiology in Epi‐ATs, whether there are epicardial preferential pathways or GPs directly provoking at the EASs.

The mechanism of the arrhythmogenicity associated with epicardial adipose tissue is still uncertain and considered multifactorial. A previous systematic review^4^ reported the possible mechanisms: inflammation, adipose infiltration, electrical remodeling, fibrosis and structure remodeling, autonomic nervous dysfunction, oxidative stress, gene expressing, local aromatase effect, and ventricular diastolic dysfunction. Nakahara et al.^10^ reported that epicardial adipose tissue was adjacent to the endocardial breakthrough of an SIAT and that the most likely mechanism of the AT was considered to be a neural reflex rather than mechanical stimulation because of the long distance between the AT focus at the right superior PV antrum and esophagus. In each Epi‐AT case, the epicardial adipose tissue was not only adjacent to the EASs and successful sites but also existed and continued between the EASs and successful sites. These facts might suggest that the hypothetic formation of epicardial preferential pathways due to structure remodeling was an epicardial adipose tissue effect. Nagashima et al.^13^ showed that the epicardial adipose tissue volumes on CT images correlated with a higher recurrence of AF after CA. In our Epi‐AT cases, no AF recurrences were ever recorded, however, their epicardial adipose tissue volumes were significantly larger than in the non‐Epi‐AT cases. Eliminating ectopic arrhythmia sources originating from epicardial sites could result in a reduction in AF recurrences, and conduction block between AT foci and endocardial breakout sites could lead to an actual PV isolation and greater durability. Zghaib et al.^21^ reported that fractionated potentials and an abnormal endocardial bipolar voltage were associated with epicardial adipose tissue as a result of atrial remodeling. In our Epi‐AT cases, fractionated potentials were recorded in 67% of the Epi‐ATs, however, an abnormal voltage was observed in only one case. That fact might suggest that the association between epicardial adipose tissue and an abnormal voltage in the case of an Epi‐AT has a different etiology from that of AF, and these findings suggested that this was one of the reasons why no AF recurrences were recorded despite the rich epicardial adipose tissue.

The acute success rate of focal AT ablation is relatively lower at 84% than that of other supraventricular tachyarrhythmias, because focal AT is sometimes an unmappable arrhythmia during endocardial catheter ablation.^22^ Knowledge of the data reported in the present study could provide therapeutic options for these unmappable and incurable focal AT cases by considering the presence of epicardial structures including epicardial adipose tissue, the vein of Marshall, and an epicardial myocardial bridge around the PVs.

## 5 LIMITATIONS

This study had several limitations. First, in four of six patients, the recognition of Epi‐ATs was during or just before the ablation procedure, we did not use drugs because of the risk of a modification of the arrhythmogenic substrate of the AT. The most striking finding was the degree to which these ATs were subject to modulation by the autonomic nervous system. However, in these patients, we were not able to evaluate the pharmacological effects with appropriate doses for blocking the adrenergic and/or cholinergic stimuli. Second, the atrial wall thickness was quite thin, such that an Epi‐AT might still mimic as a focal early site in the endocardial map which was not included in our definition of an Epi‐AT. ATs originating from epicardial foci might be much more common. Even in epicardial origins, they could be successfully ablated from the endocardial side leading to an underestimation of the Epi‐ATs. Therefore, there were no clinical features, ECG parameters including the P wave duration and morphology, or autonomic predictive features that should give a high index of suspicion of an Epi‐AT. Third, in our Epi‐AT cases, fortunately, acute success was achieved with endocardial RFCA, however, an electrical isolation including the EASs resulted in a recurrence and/or acute unsuccessful ablation. An indication for a pericardial puncture to access the epicardial atrial areas might be considered in the case of an acute unsuccessful ablation after an electrical isolation, when there is a confusing low voltage areas or fragment potentials, which could lead to inadequate endocardial mapping, and more likely to avoid vascular stenosis when RF applications to the thoracic veins would be required. Fourth, if ganglionated plexi directly provoke an AT, it is not necessarily true that the AT arose from the myocardia of epicardial sites. If their axons are distributed only in “normal” myocardia, should the origin of the ATs be considered to arise from the ganglionated plexi or “normal” myocardia? It was somewhat confusing that the definition of the Epi‐AT and Epi‐AT origins in the present study included these situations, which might mean they would be defined as an epicardial structure induced or related AT.

## 6 CONCLUSION

Six drug‐refractory ATs (12%) emerging from epicardial sites were observed out of 49 focal non‐reentrant ATs. Their foci converged around the thoracic veins including the VOM where GP rich epicardial adipose tissue was located. The epicardial adipose tissue volume of the atrium in Epi‐AT cases was significantly larger than that in non‐Epi‐AT patients, and the autonomic nerve activity directly affected the AT initiation and termination in all Epi‐AT cases. These ATs were successfully eliminated by RFCA from the endocardial side targeting AT foci or GPs, however, an electrical isolation might result in causing acute failures or recurrence.

## Supporting information


**Video S1** A Valsalva maneuver during the end‐inspiratory phase just after the 3rd inspiration in this video clip transiently suppressed a respiratory cycle‐dependent atrial tachycardia (AT‐2). Only 1 sequence of an atrial burst and some isolated ectopic beats were recorded during a Valsalva maneuver for about 15 seconds, which suppressed the AT even after 2 respiratory cycles.
